# Long-Term Leukocyte Filtration Should Be Avoided during Extracorporeal Circulation

**DOI:** 10.1155/2013/612848

**Published:** 2013-12-26

**Authors:** Jiali Tang, Kaiyu Tao, Jing Zhou, Chongwei Zhang, Lina Gong, Nanfu Luo, Lei Du

**Affiliations:** ^1^Department of Anesthesiology and Translational Neuroscience Center, West China Hospital, Sichuan University, 37 Wainan Guoxuexiang, Chengdu, Sichuan 610041, China; ^2^Department of Thoracic Cardiovascular Surgery, Affiliated No. 2 Hospital, Zhejiang University, No. 88 Jiefang Road, Hangzhou, Zhejiang 310009, China; ^3^Department of Laboratory Medicine, West China Hospital, Sichuan University, 37 Wainan Guoxuexiang, Chengdu, Sichuan 610041, China

## Abstract

Filtration during extracorporeal circulation (ECC) not only removes but also activates leukocytes; therefore, long-term leukocyte filtration may cause adverse effects. In the present study, we tested this hypothesis by priming ECC with 300 mL of canine blood and examining filtration effects in 3 groups (*n* = 6 each) during 60 min ECC. In the control group (Group C) blood was filtrated with an arterial filter for 60 min; in long-term (Group L) and short-term (Group S) groups, blood was filtrated with a leukocyte filter for 60 and 5 min. We found that about 90% of leukocytes were removed after 5 min of filtration in both Groups L and S. Although leukocyte count continued to reduce, mean fluorescent intensities of CD11/CD18, free hemoglobin, and neutrophil elastase increased in Group L and were higher than those in Groups C and S at 60 min. Leukocyte rupture, cytoplasmic leakage, and circulating naked nuclei were also found in Group L. The data support our hypothesis that long-term filtration can induce inflammation and lead to leukocyte destruction.

## 1. Introduction

Inflammatory response induced by leukocyte activation during extracorporeal circulation (ECC) is one of the major causes of organ dysfunction [1–3]. Blocking leukocyte activation may attenuate acute lung and renal injuries [4, 5]. Therefore, removing leukocytes by filtration may be beneficial to patients. However, clinical results have been controversial [6–14]. Some studies have suggested that leukocyte filters could reduce expression of CD11b/CD18 in leukocytes [6], alleviate systemic inflammatory responses [7, 8], and improve outcomes [7, 9, 10]. However, other studies have shown that leukocyte filters cannot reduce [11], and may even increase, the release of inflammatory cytokines and proteases, and thus could have a negative effect [12–14].

Leukocyte filtration activates leukocytes [14–17], which may enhance inflammatory response during ECC. Our previous *in vivo* study showed that short-term filtration effectively reduced leukocyte count, and preserved lung function better than long-term filtration. For example, neutrophil elastase (NE) and IL-8 increased during long-term filtration [18]. The results suggest that long-term application of leukocyte filtration may be contraindicated. In the present study, we tested this hypothesis with an ECC model *in vitro*.

## 2. Materials and Methods

The study was approved by the Animal Hospital Ethics Committee and all animals in the study received standard care in compliance with the “Guide for the Care and Use of Laboratory Animals.” Eighteen healthy adult hybrid dogs were provided by the Animal Experiment Center of Sichuan University for the study. Dogs (either sex, 23–35 kg BW) were anesthetized with sodium pentobarbital (25 mg/kg). After heparinization (3 mg/kg), 300 mL of blood was harvested from the left femoral artery to a blood bag. Meanwhile, 6% hydroxyethyl starch 130/0.4 (Fresenius Kabi, Germany) was infused from the femoral vein to maintain blood pressure.

### 2.1. ECC Protocol

A closed ECC loop was established with a blood storage device (Xi-Jing Medical Ltd, Xi'an, China) and a regular arterial filter (Xi-Jing Medical Ltd, Xi'an, China) or a leukocyte filter (Heart-Care System, Separator Haemo-Technology Beijing Co Ltd. China), a roller pump (StÖckert II, Munich, Germany), and tubing. The ECC loop was primed with 300 mL of preserved blood harvested above and 300 mL hydroxyethyl starch 130/0.4. The circulating flow rate was set at 1 L/min, and ECC lasted for 60 min in all groups.

Our pilot study showed that the leukocyte count decreased sharply with low levels of NE after 5 min. Therefore, 5 min was used for “short-term” test in this study. In clinical trials, however, a leukocyte filter is used throughout ECC, often longer than 60 min. Therefore, 60 min was used for “long-term” test.

Established ECCs were divided randomly into 3 groups (*n* = 6 in each group). Blood from the control group (Group C) was filtrated throughout ECC with a standard arterial filter. Blood from Groups L (long-term) and S (short-term) was filtrated with a leukocyte filter for 60 and 5 min (the filter was bypassed in the remaining 55 min). Blood samples were obtained at 0, 5, 30, and 60 min of ECC for indexes analyses.

### 2.2. Analysis of Blood Cell, NE, and fHb

Cell count and differentiation were performed by an automatic blood cell analyzer (MINDRAY, BC-3000, China) [19]. For measurements of NE and free hemoglobin (fHb) levels, blood samples were centrifuged for 15 minutes at 1000 ×g and 4°C. The supernatant was then removed and stored at −80°C until it was analyzed. NE levels were determined by Enzyme Linked Immunoadsorbent Assay (NE ELISA kits, Uscn Life Science Inc. Wuhan, China), according to the manufacturer's instruction. The levels of fHb were determined by the orthotolidine method [20].

### 2.3. CD11/CD18 Expression

For determination of expression of CD11/CD18 on leukocyte, red blood cells were lysed with ammonium chloride solution, and acquired leukocytes were resuspended in 100 *μ*L of phosphate-buffered saline. Nonspecific antibody binding was blocked with FcR-blocking reagent (Miltenyi Biotec) for 20 min before staining with conjugated antibodies. Immunofluorescent cell staining was performed with Rat anti dog CD11/CD18:FITC (Clone: YKIX490.6.4, AbD Serotec Co., Ltd., Oxford Kidlington, UK). Isotype: IgG2c (AbD Serotec Co., Ltd., Oxford Kidlington, UK) served as a negative control. Data acquisition was performed on a FACSAria cytometer equipped with FACS Diva 5.0 software (BD) and analyzed by Flowjo software (Tree Star) [4, 21].

### 2.4. Morphology of Circulating Nucleated Cells

Circulating integral leukocytes were determined as we described previously [19]. Briefly, red blood cells were lysed; the sample was centrifuged at 302 ×g for 5 min and washed with PBS 3 times. Nucleated cell suspensions were smeared and stained with Wright's stain. One hundred nucleated cells per slide were counted under a microscope. Only naked nuclei that stained blue were calculated.

### 2.5. Ultrastructure of Filer Membrane

At the end of experiment, the membrane of the leukocyte filter was taken for transmission electron microscopy as reported previously [3, 19]. Samples were fixed in 3% glutaraldehyde at 4°C overnight and then fixed with 1% osmium tetroxide, dehydrated with acetone, and embedded in Epon812. The ultrathin sections were double stained with uranyl acetate and lead citrate and examined under a transmission electron microscope (H-600IV, Hitachi, Osaka, Japan).

### 2.6. Statistics

Data were analyzed by SPSS 16.0 (SPSS, Inc, Chicago, Ill). Quantitative data were expressed by the mean ± SD, and one-way ANOVA with Student-Newman-Keuls test was used to compare differences among the 3 groups. Segmental data were expressed as a percentage, and the differences between the 3 groups were compared using the chi-square test. A value of *P* < 0.05 was considered statistically significant.

## 3. Results

### 3.1. Long-Term Filtration Removed and Activated Leukocytes

**Figure 1:**
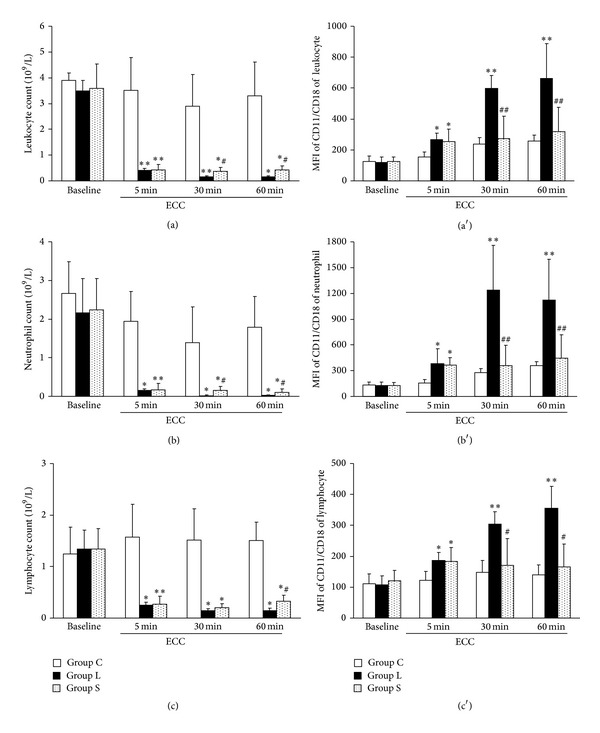
Long-term filtration during extracorporeal circulation (ECC) not only removes but also activates leukocytes. A closed ECC loop was established with a regular arterial filter (Group C) or a leukocyte filter (Group S or L, *n* = 6 in each group). The ECC was primed with 300 mL of blood. Blood in Group S was filtrated for 5 min, then the leukocyte filter was bypassed, while blood in Group L was filtrated throughout the 60 min experiment. Leukocytes (a), neutrophils (b), and lymphocytes (c) were reduced after 5 min of filtration in groups L and S. However, mean fluorescent intensities (MFI) of CD11/CD18 of leukocytes (a′), neutrophils (b′), and lymphocytes (c′) were significantly higher in Group L than in groups C and S. *P < 0.05 and **P < 0.01 versus control; and ^#^ *P* < 0.05 and ^##^ *P* < 0.01 versus Group L.

Leukocyte counts were similar among the 3 groups before ECC and decreased by 90% after 5 min of filtration in groups L and S (Figure 1(a)). Then, it continued to decrease throughout 60 min of ECC in Group L.

Mean fluorescent intensities (MFIs) of CD11/CD18 on leukocyte increased significantly in both groups L and S compared with that in Group C at 5 min ECC (Figure 1(a′), *P* < 0.01). Thereafter, MFIs of CD11/CD18 in the leukocyte increased sharply in group L, while it increased gradually in groups C and S. Neutrophils and lymphocytes, and expression of CD11/CD18 had a similar pattern (Figures 1(b), 1(c), 1(b′), and 1(c′)).

**Figure 2:**
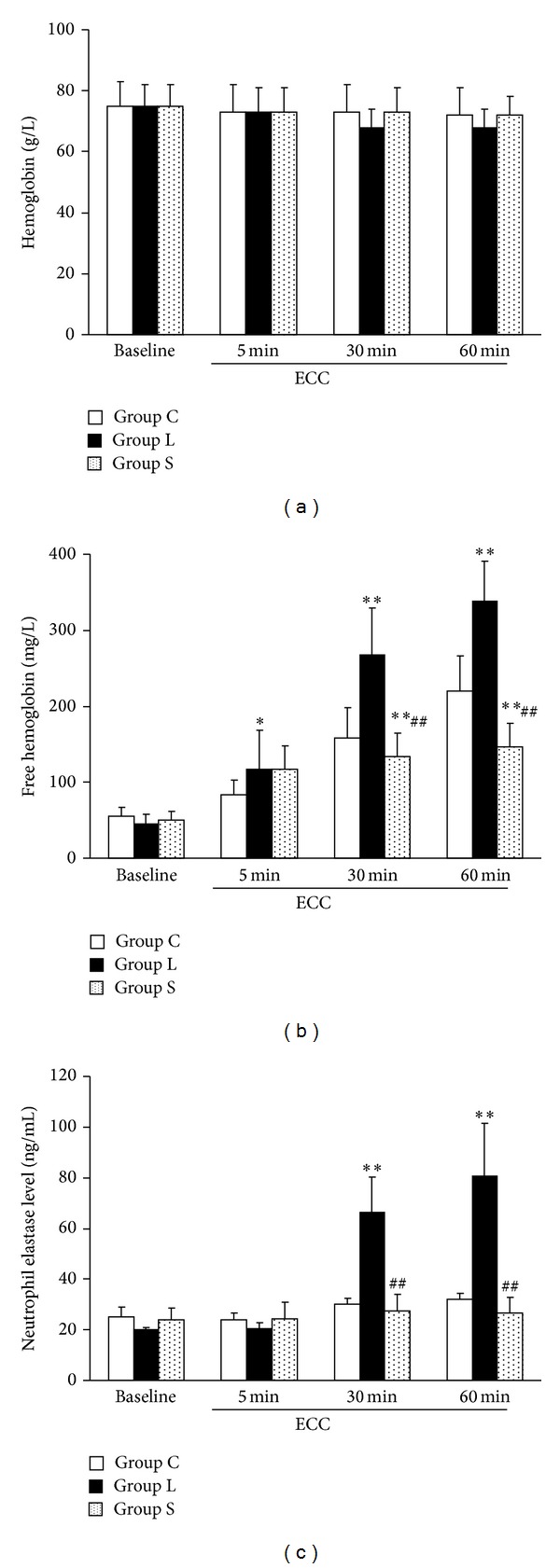
Long-term filtration causes erythrocyte injury and neutrophil elastase release. Groups were divided as those in Figure 1 (*n* = 6 in each group). Hemoglobin levels were similar in all groups during experiments (a). Both of free hemoglobin (b) and neutrophil elastase (c) levels were higher in Group L than in Groups C and S after 30 min of ECC. Free hemoglobin level was also higher in Group C than in Group S. *P < 0.05 and **P < 0.01 versus control; and ^#^ *P* < 0.05 and ^##^ *P* < 0.01 versus Group L.

NE is released most from the activated leukocyte, and induces an inflammatory response in blood and tissues. To confirm leukocyte activation, plasma NE was determined. NE level in Group L increased significantly at 30 min of filtration, and continued to increase to 60 min (Figure 2(c)). It was significantly higher in Group L than groups S and C.

### 3.2. Long-Term Filtration Accelerated Erythrocyte Injury

To determine whether erythrocyte destruction is associated with activated neutrophils during ECC, fHb levels in plasma were measured. Hemoglobin (a) and fHb (b) levels were similar among the 3 groups before ECC (Figure 2). The fHb level increased during ECC, and was significantly higher in Group L than in the other 2 groups at 30 and 60 min (*P* < 0.01). Furthermore, the fHb level was lower in Group S than in Group C.

### 3.3. Leukocytes Were Ruptured by Filtration

**Figure 3:**
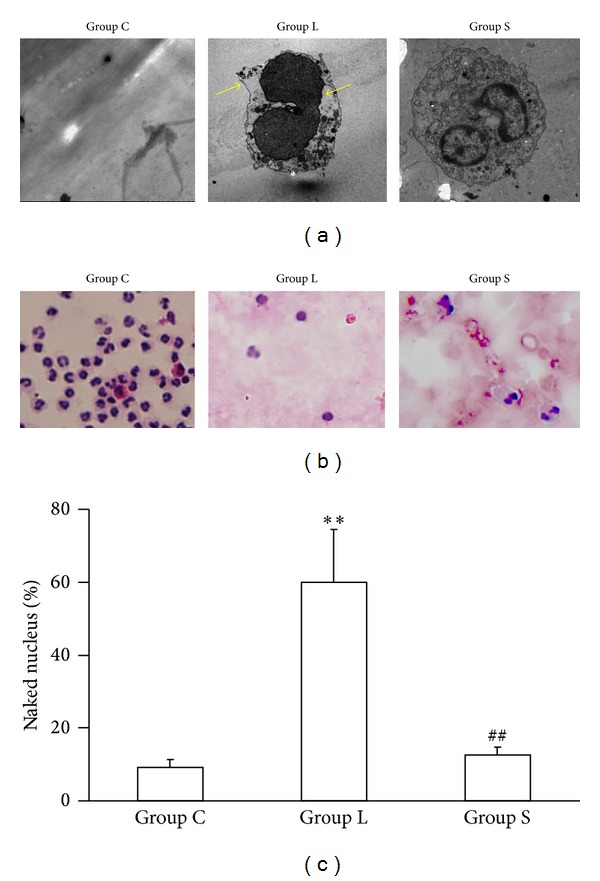
Leukocyte is ruptured by long-term filtration. Groups were the same as in Figure 1 (*n* = 6 in each group). (a) Transmission electron microscope of leukocyte. Cellular membrane rupture and cytoplasmic leakage (yellow arrow) were found in Group L. (b) Wright's stain of nucleated cell. (c) Percentage of naked leukocyte nuclei were higher in Group L than these in groups C and S. **P < 0.01 versus Group C; ^##^ *P* < 0.01 versus Group L.

Wright's stain and transmission electron microscopy analyses showed significant leukocyte rupture in Group L (Figure 3), indicated by cellular membrane rupture and cytoplasmic leakage ((a), yellow arrow). Accordingly, the percentage of naked nuclei in blood was highest in Group L.

## 4. Discussions

Our study is the first *in vitro* approach that focused on filtration induced inflammation. Using the extracorporeal circulation (ECC) model *in vitro*, we clearly showed that effectiveness of leukocyte filtration depends on exposure time during ECC. Short-term use of a leukocyte filter reduced circulating leukocytes; with continued usage, leukocytes that adhered to the filter membrane disrupted, leading to leakage of protease, inflammatory reaction, and tissue destruction. Our results may explain why leukocyte depletion could not improve the outcomes in some clinical studies [12–14].

Studying the effect of filtration on inflammation is difficult *in vivo*, because inflammatory products are processed by the immune system. For example, naked nuclei in the circulation would be engulfed. Therefore, the *in vitro* model may be a necessary approach. In this *in vitro* model, circulating leukocytes were significantly reduced with a 5 min short-term filtration (about 5–7 circles). The leukocyte filter was then bypassed, and leukocytes adhered to the membrane were no longer exposed to the circulation. Our results support a short-term usage of the filter because the concentration of fHb was lower in Group S than in Group C. This is in agreement with several clinic trials. Morioka et al. [22] and Chiba et al. [23] found leukocyte depletion before ECC significantly attenuated both myocardial and lung injuries induced by cardiopulmonary bypass. In patients with systemic inflammatory response syndrome, a short-term leukocyte depletion significantly improved organ function and outcomes [24]. In patients with chronic obstructive pulmonary diseases, leukocyte filtration at early reperfusion (30 min) improved oxygenation and shortened intubation and ICU and hospital times [25].

Efficiency of a filter is determined by membrane area and filtration time. The filtration would reach its maximal capacity by prolonging filtering time. Then, the number of circulating leukocytes would not change with continued use of the filter; however, damaging effects on leukocytes develop, which compromises the beneficial effect of the filter. An optimal length of time for filter use should be available.

Previous studies have observed that leukocyte filtration did not inhibit, but activated leukocytes [14–17]. This is consistent with our results that CD11/CD18 expression on leukocytes was significantly upregulated with long-term filtration, which induces leukocyte sequestration in the tissues. Furthermore, leukocytes adhered to the membrane were elongated and ruptured under the transmission electron microscope after long-term filtration. This result was confirmed by a high level of circulating naked nucleus count and neutrophil elastase. Taken together, our results indicate that long-term filtration during ECC induces leukocyte rupture.

Activated leukocytes induce tissue injury via obstruction of microcirculation and release of proteases, such as neutrophil elastase. In the present study, neutrophil elastase and free hemoglobin increased in Group L significantly, suggesting that long-term usage of leukocyte filtration exerts deleterious side effects. This statement can be verified by the fact that aprotinin, a serine proteases inhibitor, significantly improves lung function and reduces the incidence of atrial fibrillation in patients treated with a leukocyte filter [26].

Although our results showed that short-term filtration may effectively inhibit inflammatory reactions and reduce erythrocyte destruction, whether it would improve patient outcomes requires further *in vivo* studies.

In conclusion, our results clearly show that long-term filtration can induce leukocyte destruction and evoke inflammatory reactions and should be avoided.
